# Essential role for acid sphingomyelinase-inhibited autophagy in melanoma response to cisplatin

**DOI:** 10.18632/oncotarget.8735

**Published:** 2016-04-14

**Authors:** Davide Cervia, Emma Assi, Clara De Palma, Matteo Giovarelli, Laura Bizzozero, Sarah Pambianco, Ilaria Di Renzo, Silvia Zecchini, Claudia Moscheni, Chiara Vantaggiato, Patrizia Procacci, Emilio Clementi, Cristiana Perrotta

**Affiliations:** ^1^ Department for Innovation in Biological, Agro-food and Forest Systems (DIBAF), Università degli Studi della Tuscia, Viterbo, Italy; ^2^ Department of Biomedical and Clinical Sciences “Luigi Sacco” (DIBIC), Università degli Studi di Milano, Milano, Italy; ^3^ Scientific Institute IRCCS Eugenio Medea, Bosisio Parini, Italy; ^4^ Present address: Division of Experimental Oncology, San Raffaele Scientific Institute, Milano, Italy; ^5^ Unit of Clinical Pharmacology, National Research Council-Institute of Neuroscience, University Hospital “Luigi Sacco”, Milano, Italy; ^6^ Present address: Department of Oncology, Università degli Studi di Torino and Laboratory of Neurovascular Biology, Candiolo Cancer Institute, Candiolo, Italy; ^7^ Department of Biomedical Sciences for Health (SCIBIS), Università degli Studi di Milano, Milano, Italy

**Keywords:** A-SMase, melanoma, autophagy, mTOR, chemo-resistance

## Abstract

The sphingolipid metabolising enzyme Acid Sphingomyelinase (A-SMase) has been recently shown to inhibit melanoma progression and correlate inversely to tumour grade. In this study we have investigated the role of A-SMase in the chemo-resistance to anticancer treatmentusing mice with melanoma allografts and melanoma cells differing in terms of expression/activity of A-SMase. Since autophagy is emerging as a key mechanism in tumour growth and chemo-resistance, we have also investigated whether an action of A-SMase in autophagy can explain its role. Melanoma sensitivity to chemotherapeutic agent cisplatin in terms of cell viability/apoptosis, tumour growth, and animal survival depended directly on the A-SMase levels in tumoural cells. A-SMase action was due to inhibition of autophagy through activation of Akt/mammalian target of rapamycin (mTOR) pathway. Treatment of melanoma-bearing mice with the autophagy inhibitor chloroquine restored sensitivity to cisplatin of tumours expressing low levels of A-SMase while no additive effects were observed in tumours characterised by sustained A-SMase levels. The fact that A-SMase in melanomas affects mTOR-regulated autophagy and plays a central role in cisplatin efficacy encourages pre-clinical testing on the modulation of A-SMase levels/activity as possible novel anti-neoplastic strategy.

## INTRODUCTION

Melanoma is a high-grade, poorly differentiated malignant tumour of the melanocytes that hesitates frequently in the metastatic stages and accounts for most of the skin cancer related deaths [1–3]. Whereas thin melanomas have an excellent prognosis after sufficient surgical treatment, melanoma disease in advanced stages is still a therapeutic challenge despite the large number of chemotherapeutic regimens so far developed [4–8]. Single drug chemotherapy is in many cases ineffective and combinations of chemotherapeutic drugs have shown response rates only marginally higher, and at the cost of systemic toxicity [4–8]. New targeted therapies and immunotherapies have been recently approved; these show better efficacy and have supplanted chemotherapy as first- and second-line therapy [7–13]. Since melanoma cells eventually become resistant also to these novel therapies, the quest for novel, more effective and possibly less toxic approaches is still open.

Autophagy, a homeostatic proteolytic system of the cell with a role in the removal of proteins, aggregates and organelles within the lysosome [14–17], is a crucial determinant of the melanoma sensitivity to chemotherapeutic drugs [18–25]. The mechanism regulating autophagy impact on drugs efficacy still needs to be fully clarified. Such an information would help designing new strategies for melanoma treatment.

The sphingolipid metabolising enzyme Acid Sphingomyelinase (A-SMase) contributes to apoptotic death of tumour cells and is important in determining tumour sensitivity/resistance to antineoplastic treatments, including cisplatin, one of the most potent agents with clinical activity against solid tumours [26–36]. We recently found that A-SMase actually contributes to determine the malignant phenotype of melanoma cells *in vitro* and *in vivo* in terms of tumour progression, invasiveness and metastatic capacity. In addition, A-SMase levels of expression correlated with melanoma grade in human biopsies [37, 38]. Tumours expressing low A-SMase levels displayed high levels of inflammatory factors and an immune-suppressive/pro-tumoural microenvironment. Overexpression of A-SMase induced a high recruitment at the tumour site of effector immune cells with an anti-tumoural function [37, 38]. Interestingly A-SMase and sphingolipids appear to regulate autophagy, especially the process of lysosome trafficking and fusion [39, 40].

In this study we have investigated the role of A-SMase on autophagy using mice with melanoma allografts and mouse and human melanoma cells differing in terms of expression/activity of A-SMase. We found that the inhibition of autophagy by A-SMase determines tumour response to cisplatin in a mechanism involving activation of the Akt/mammalian target of rapamycin (mTOR) pathway. These results support the importance of A-SMase as a therapeutic prospective target in melanoma progression.

## RESULTS

### Chemo-resistance to cisplatin is inversely proportional to A-SMase levels

**Figure 1:**
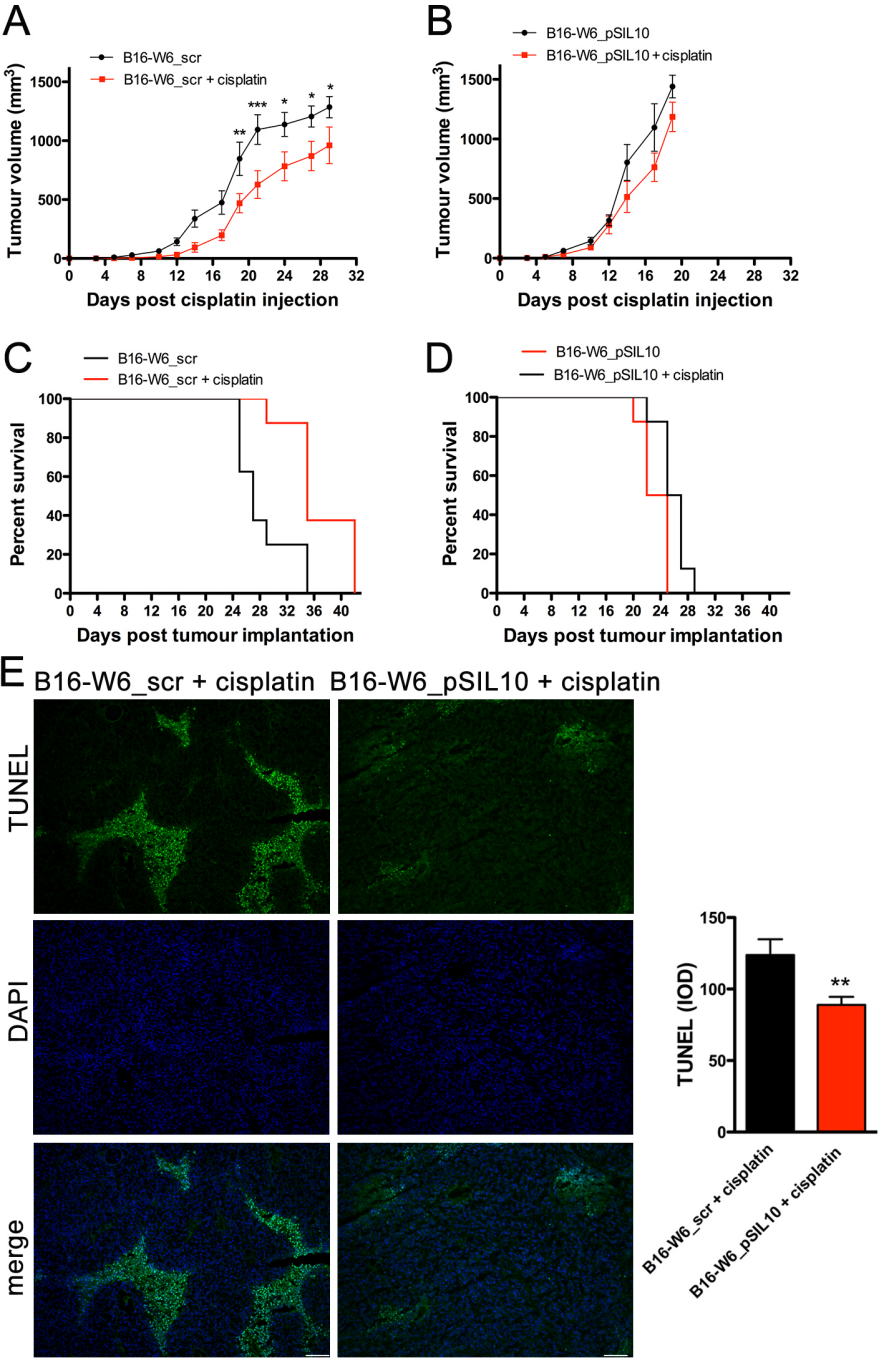
Chemo-resistance to cisplatin *in vivo* is inversely proportional to A-SMase levels C57BL/6 mice (*n* = 8) were injected in the right flank with B16-W6_scr (A and C) and B16-W6_pSIL10 (B and D) cells. When tumours where palpable cisplatin (4 mg/kg) or vehicle was injected intraperitoneally three times every other day. **A.**-**B.** Tumour growth was monitored by measuring tumour volume (mm^3^) every 2-3 days. Statistical significance *p < 0.05, **p < 0.01, and ***p < 0.001 *vs* untreated mice. **C.**-**D.** Kaplan-Meier survival curve of animals. **E.** Left panels: representative fluorescence micrographs of TUNEL and DAPI staining in B16-W6_scr and B16-W6_pSIL10 tumours (500 mm^3^ volume) excided from mice injected with cisplatin. Scale bar: 100 μm; Right panel: quantitative analysis of TUNEL staining (*n* = 3). Values are expressed as integrated optical density (IOD). Statistical significance **p < 0.01 *vs* B16-W6_scr + cisplatin.

To assess whether A-SMase is involved in *in vivo* chemo-resistance of melanoma to cisplatin we generated highly tumourigenic mouse B16 melanoma allografts. To this end we used two cell clones B16-W6_scr and B16-W6_pSIL10 already characterised for tumourigenic potential and A-SMase activity: B16-W6_pSIL10 shows significantly lower expression/activity of A-SMase than B16-W6_scr and significantly higher tumour-inducing potential [38]. Allografts were obtained by injecting sub-cutaneously either cell clone. When tumour was established, cisplatin was injected intraperitoneally. Cisplatin induced a significant decrease in the tumour volume of B16-W6_scr transplants but not of B16-W6_pSIL10 tumours (Figure 1A-1B). Consistently, cisplatin significantly imcreased the survival of B16-W6_scr-injected mice (median of survival: B16-W6_scr = 27 days, B16-W6_scr + cisplatin = 35 days), while only marginally increasing that of B16-W6_pSIL10-injected mice (median of survival: B16-W6_pSIL10 = 23.5 days, B16-W6_pSIL10 + cisplatin = 26 days) (Figure 1C-1D). Cisplatin-treated B16-W6_pSIL10 tumours showed significantly less DNA fragmentation, detected *via* TUNEL staining, than B16-W6_scr tumours (Figure 1E), in keeping with the notion that A-SMase-dependent chemo-resistance to cisplatin may involve sensitivity to apoptosis [35].

**Figure 2:**
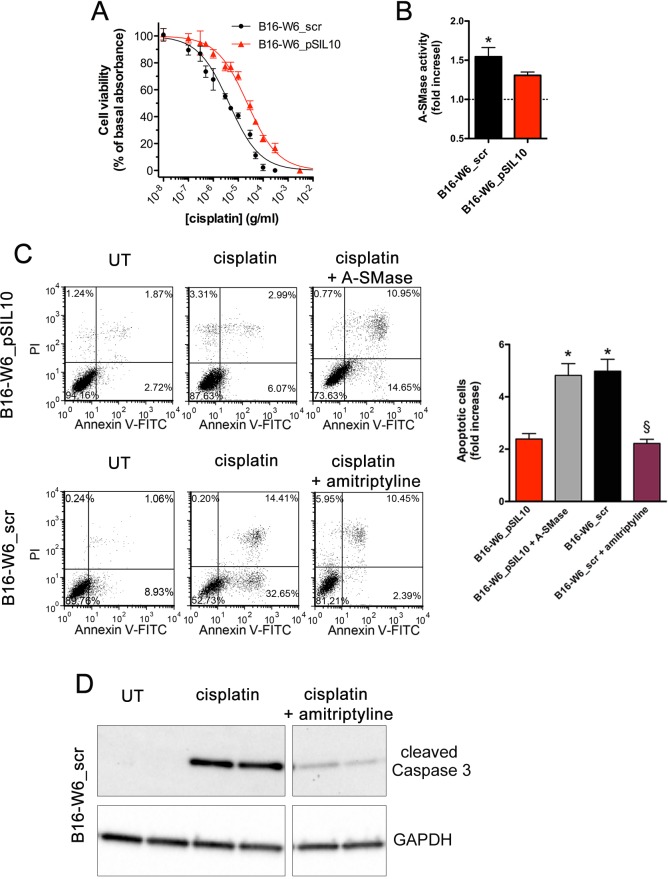
Chemo-resistance to cisplatin *in vitro* is inversely proportional to A-SMase levels **A.** Dose-response curves of the effects of cisplatin on the viability of B16-W6_scr and B1-W6_pSIL10, as measured by the MTT assay (*n* = 10). **B.** Measurement of A-SMase activity in B16-W6_scr and B1-W6_pSIL10 cells treated with cisplatin (10 μg/ml, 16 h). Activity was expressed as fold increase of untreated cells (*n* = 5). Statistical significance *p < 0.05 *vs* untreated B16-W6_scr. **C.** B1-W6_pSIL10 and B16-W6_scr were cultured for 16 h in the absence (UT: untreated) or in the presence of cisplatin (10 μg/ml). B1-W6_pSIL10 and B16-W6_scr were also treated with human A-SMase (2.0 units/ml) or amitriptyline (5 μM, 1 h before cisplatin treatment), respectively. Left panels: representative dot plots of Annexin V-FITC/PI staining; Right panel: apoptosis quantification expressed as fold increase of total apoptotic cells (Annexin V^+^/PI^−^ and Annexin V^+^/PI^+^ cells) compared to their respective UT controls (*n* = 8). Statistical significance *and § *p* < 0.05 *vs* B1-W6_pSIL10 and B16-W6_scr, respectively. **D.** Western blot analysis of cleaved Caspase 3 expression in B16-W6_scr cells cultured for 16 h in the absence (UT) or in the presence of cisplatin (10 μg/ml) and cisplatin + amitriptyline (5 μM, 1 h before cisplatin treatment). GAPDH was used as the internal standard. The images are representative of results obtained from three experiments.

We then examined the effects of cisplatin on the viability of melanoma cells. B16-W6_scr and B16-W6_pSIL10 cells were cultured for 16 h in the absence or presence of increasing concentrations of cisplatin, before an MTT assay. As shown in Figure 2A, cisplatin caused a concentration-dependent reduction of basal absorbance with a significantly lower potency (0.8 log units; *p* < 0.0001) in B16-W6_pSIL10 (pEC_50_: 4.6 ± 0.03) *versus* B16-W6_scr (pEC_50_: 5.4 ± 0.03) cells. Of importance cell viability correlated with A-SMase activity. Cisplatin at 10 μg/ml (a concentration around EC_50_) significantly increased A-SMase activity in B16-W6_scr cells but not in B16-W6_pSIL10 cells (Figure 2B). Apoptosis was then analysed by flow cytometry of Annexin V^+^/propidium iodide (PI)^−^ and Annexin V^+^/PI^+^ cell fractions, markers of early and late apoptotic stages, respectively. The apoptotic effects of cisplatin were significantly increased in B16-W6_scr when compared to B16-W6_pSIL10 cells (Figure 2C). The exogenous addition of human A-SMase (2.0 units/ml) [35, 38] to B16-W6_pSIL10 cells restored in full their sensitivity to cisplatin. Since A-SMase is an acidic pH optimum SMase, which has been shown to enter cells *via* endocytosis becoming active in acidic compartments, we also treated mouse melanoma cells with bacterial A-SMase, which activity has neutral pH optima and is used to hydrolyse sphingomyelin localised at the plasma membrane [41]. Exogenously added bacterial A-SMase (0.3 units/ml) [42] in B16-W6_pSIL10 cells had no effect on cisplatin-induced apotosis, thus indicating an intracellular action of A-SMase instead that a mechanism confined only to cell (Supplementary Figure 1). Pharmacological inhibition of A-SMase by amitriptyline (5 μM, 1 h before cisplatin treatment), a widely used lysosomotropic molecule that induces loss of A-SMase [43], significantly decreased the percentage of cisplatin-induced B16-W6_scr apoptotic cells at levels comparable to B16-W6_pSIL10 cells (Figure 2C), further indicating that low A-SMase inhibits cisplatin-induced melanoma cell death. These data were then confirmed by western blot experiments with an antibody that binds to cleaved (active) Caspase 3, an hallmark of apoptosis. In particular, as shown in Figure 2D, B16-W6_scr treated with cisplatin expressed higher levels of Caspase 3 activity when compared to untreated cells. Of notice, amitriptyline decreased the expression of cleaved Caspase 3 induced by cisplatin. Similar results were obtained in human melanoma cell lines expressing different levels of A-SMase protein and enzymatic activity (Supplementary Figure 2A). In particular, MTT assay demonstrated that Det-mel cells, characterised by low A-SMase [38], exhibited a significant rightward shift (0.7 log units; *p* < 0.0001) of the cisplatin concentration-dependent curve (pEC_50_: 4.5 ± 0.08) when compared with MRS3 cells (pEC_50_: 5.2 ± 0.07), characterised by high A-SMase [38]. Cisplatin at 10 μg/ml did not induce apoptosis in low A-SMase Det-mel cells while the administration of exogenous human A-SMase increased significantly the cell sensitivity to the chemotherapeutic drug (Supplementary Figure 2B).

### A-SMase levels correlate inversely with autophagy

**Figure 3:**
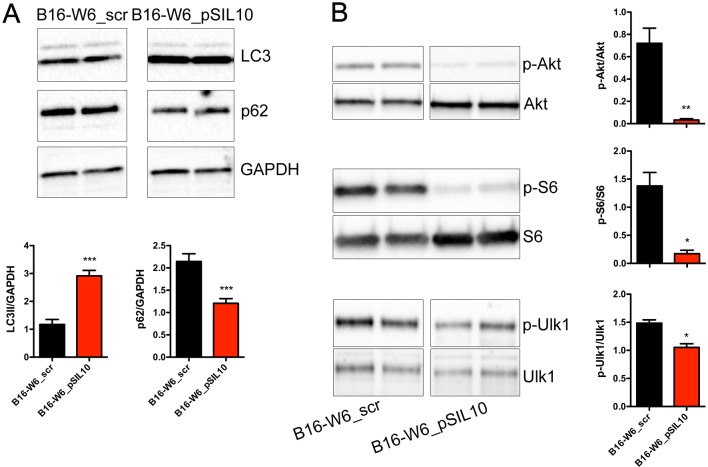
A-SMase levels affect autophagic process *in vitro* **A.** Upper panels: representative western blot analysis of LC3 lipidation and p62 expression in B16-W6_scr and B1-W6_pSIL10 cells. GAPDH was used as the internal standard; Lower panels: densitometric analysis of lipidated LC3 (LC3 II) and p62 expression (*n* = 6-7). **B.** Left panels: representative western blot analysis of phosphorylated Akt, S6 and Ulk1 in B16-W6_scr and B1-W6_pSIL10 cells. The total Akt, S6 and Ulk1 were used as internal standard; Right panels: densitometric analysis of phosphorylation levels of Akt, S6, and Ulk1 (*n* = 4-8). Statistical significance *p < 0.05, **p < 0.01, and ***p < 0.001 *vs* B16-W6_scr.

Autophagy contributes to the anticancer efficacy of chemotherapy in different types of cancer, including melanoma [18–22]. We investigated the relationship of A-SMase and autophagy in our model; autophagy was assessed by measuring LC3 and p62 protein levels by western blot experiments. LC3 is recruited from the cytosol and associates with the phagophore early in autophagy, while p62 is a cargo of ubiquitinated proteins that is degraded lately during the autophagic process and accumulates in the presence of defective autophagy [14, 16, 17]. As shown in Figure 3A, formation of lipidated LC3 (the faster migrating band is indicative of completed autophagosomes) and reduction of p62 levels was higher in B16-W6_pSIL10 than in B16-W6_scr. Autophagy is inhibited by mTOR a protein activated by Akt. mTOR increases (directly and indirectly) the phosphorylation of autophagy controlling proteins including the S6 ribosomal protein (S6) [14, 16, 17, 44] and Ulk1 [45]. We found that phosphorylated levels of Akt in B16-W6_pSIL10 were significantly lower than B16-W6_scr cells and accompanied by a lower phosphorylation levels of S6 and Ulk1 (Figure 3B).

**Figure 4:**
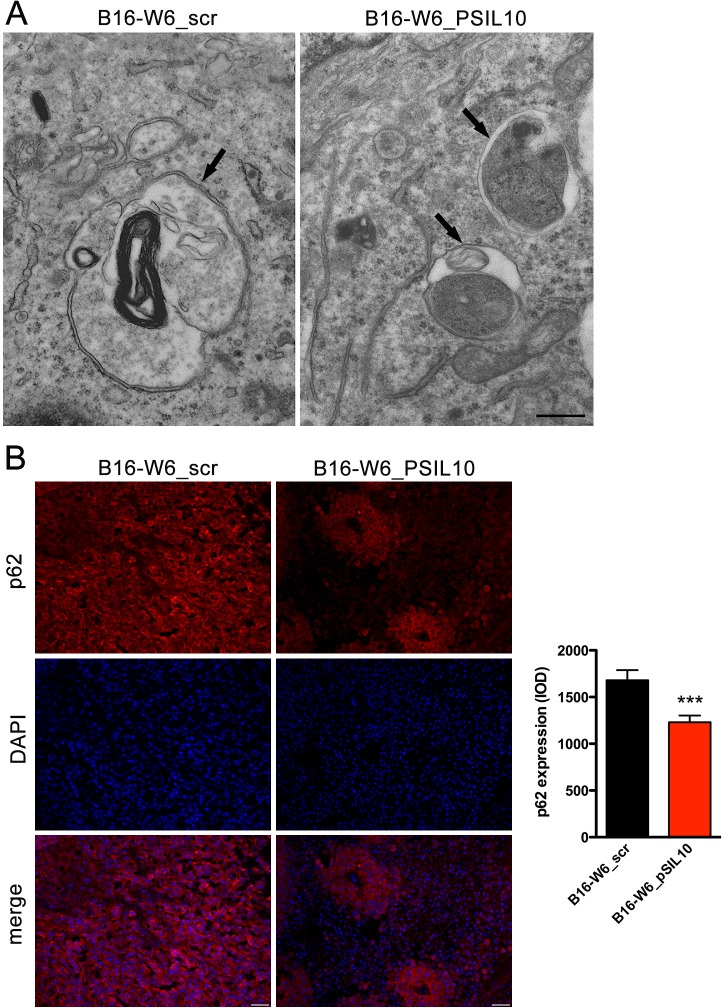
A-SMase levels affect autophagic process *in vivo* C57BL/6 mice were injected in the right flank with B16-W6_scr and B16-W6_pSIL10 cells; tumours were then resected when they reached the volume of 500 mm^3^. **A.** Transmission electron microscopy showing autophagosomes (black arrows) in B16-W6_scr and B16-W6_pSIL10 tumours. At the lower right of the B16-W6_pSIL10 micrograph a site of close vicinity between the membranes of endoplasmic reticulum and mitochondrion, i.e. MAM, can be seen. The images are representative of results obtained from three experiments. Scale bar = 0.5 μm. **B.** Left panels: representative fluorescence micrographs of p62 and DAPI staining in B16-W6_scr and B16-W6_pSIL10 tumours. Scale bar = 50 μm; Right panel: quantification of p62 immunofluorescence staining (*n* = 3). Values are expressed as integrated optical density (IOD). Statistical significance ***p < 0.001 *vs* B16-W6_scr.

The inverse correlation of A-SMase levels and autophagy was confirmed further in melanoma allografts. Ultrastructural analysis of tumours by transmission electron microscopy revealed the presence of autophagic vacuoles, such as autophagosomes [14–17, 46]. They appeared as double or single (rarely)-membrane bounded structures sometimes containing small compartments or undigested mitochondria, fragments of endoplasmic reticulum and multilamellar structures (Figure 4A). Membrane tethering sites during autophagy activation is driven by inter-organellar micro-domains among which are mitochondria-associated endoplasmic reticulum membranes (MAMs) [17, 47]. Of notice, MAMs were detected in B16-W6_pSIL10 tumours but not in B16-W6_scr. In line with these results, immunocytochemical experiments showed significant lower levels of p62 staining in B16-W6_pSIL10 tumours when compared to B16-W6_scr tumours (Figure 4B), indicative of an increased autophagic process.

### Autophagy down-regulation by A-SMase explains the response of tumours to chemotherapy with cisplatin

**Figure 5:**
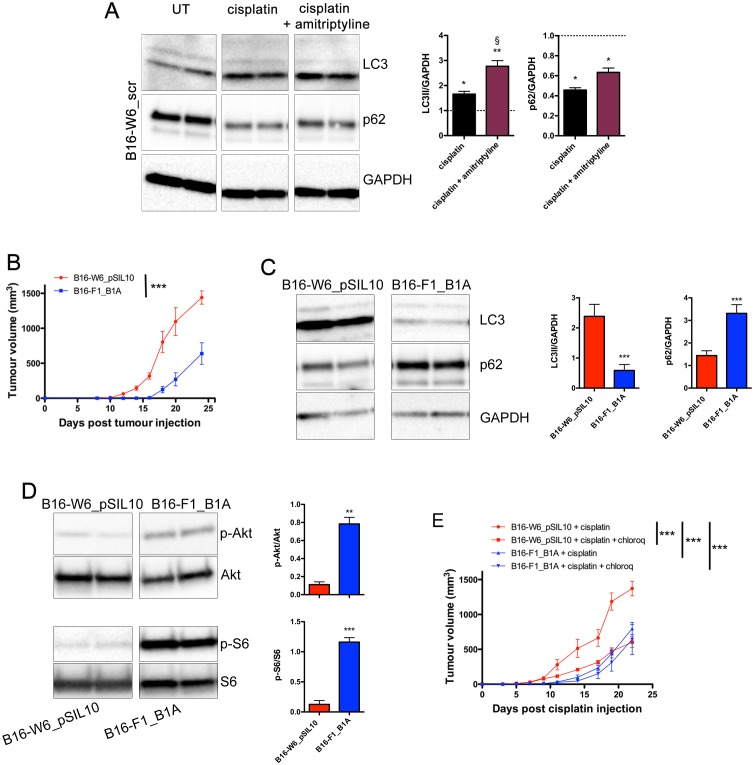
A-SMase levels, authophagy, and chemo-resistance to cisplatin **A.** Left panels: representative western blot analysis of LC3 lipidation and p62 expression in B1-W6_scr cells cultured for 16 h in the absence (UT: untreated) or in the presence of cisplatin (10 μg/ml) and cisplatin + amitriptyline (5 μM, 1 h before cisplatin treatment). GAPDH was used as the internal standard; Right panels: densitometric analysis of lipidated LC3 (LC3 II) and p62 expression measured as fold change compared to their respective UT controls (*n* = 3). Statistical significance *p < 0.05 and **p < 0.01 *vs* UT; § p < 0.05 *vs* B1-W6_scr + cisplatin. **B.** C57BL/6 mice (*n* = 7) were injected in the right flank with B16-W6_pSIL10 and B16-F1_B1A cells. Tumour growth was monitored by measuring tumour volume (mm^3^) every 2-3 days. Statistical significance ***p < 0.001. **C.** Left panels: representative western blot analysis of LC3 lipidation and p62 expression in B1-W6_pSIL10 and B16-F1_B1A cells. GAPDH was used as the internal standard; Right panels: densitometric analysis of lipidated LC3 (LC3 II) and p62 expression (*n* = 7). **D**. Left panels: representative western blot analysis of phosphorylated Akt and S6 in B1-W6_pSIL10 and B16-F1_B1A cells. The total Akt and S6 were used as internal standard; Right panels: densitometric analysis of phosphorylation levels of Akt and S6 (*n* = 5-7). Statistical significance *p < 0.05, **p < 0.01, and ***p < 0.001 *vs* B16-W6_ pSIL10. **E.** B1-W6_pSIL10 and B16-F1_B1A allografts were established as in A (*n* = 7). When tumours where palpable cisplatin alone (4 mg/kg, intraperitoneally) and cisplatin + chloroquine (30 mM, in the peritumoural area) were injected and tumour growth monitored. Statistical significance ***p < 0.001.

Recently, cisplatin was shown to induce an increase of lipidated LC3 and autophagosome formation in melanoma cells [48]. We investigated whether A-SMase-induced sensitivity to chemotherapy depended on the regulation of autophagy. B16-W6_scr treated with cisplatin (16 h, 10 μg/ml) expressed higher levels of lipidated LC3 and lower levels of p62 when compared to untreated cells (Figure 5A). The pharmacological inhibition of A-SMase with amitriptyline (5 μM, 1 h before cisplatin treatment) further increased lipidated LC3 levels after cisplatin while p62 expression was not affected.

To better support a critical role of A-SMase and autophagy in chemo-resistance, *in vivo* experiments were then performed inducing allografts with B16-W6_pSIL10 and B16-F1_B1A tumour cells; the latter representing an A-SMase over-expressing melanoma model [37]. In both models A-SMase expression is stable during tumour growth [37, 38]. Consistent with the inhibitory role of A-SMase in melanoma progression [37, 38] B16-F1_B1A tumours grew more slowly than B16-W6_pSIL10 tumours (Figure 5B) and exhibited significant lower formation of lipidated LC3 (Figure 5C), and up-regulated levels of p62 (Figure 5C) and phospho-Akt/S6 (Figure 5D). The role of autophagy in melanoma progression was then assessed by the contemporary injection of tumour bearing mice with cisplatin and chloroquine, which inhibits autophagy [18, 49–53]. As shown in Figure 5E, when both compounds were administered, B16-W6_pSIL10 growth-rate was significantly reduced. Of interest, B16-F1_B1A tumours administered with cisplatin, both in the absence and in the presence of chloroquine, displayed similar growth-rate which is also comparable to the growth-rate observed in the cisplatin and chloroquine-treated B16-W6_pSIL10 transplants.

## DISCUSSION

A-SMase plays fundamental roles in tumour pathogenesis and in their response to treatment in different types of cancer [27, 29–35, 40, 54]. The present study reveals for the first time that sensitivity to cisplatin correlates with the expression/activity of A-SMase and is mediated by the intracellular action of this enzyme: an inhibitory effect on autophagy which parallels the activation of apoptosis.

Melanoma cells expressing low A-SMase displayed marked chemo-resistance to cisplatin in terms of cell viability, tumour growth and median survival of transplanted animals accompanied by reduced cisplatin-induced apoptosis; over-expression of A-SMase enhanced the antineoplastic effect of irradiation in melanomas [34]. Changes in A-SMase activity/levels lead to a dysregulation of the autophagy [40]. In particular, A-SMase seems to play a role in the autophago-lysosomal degradation and an imbalance in A-SMase activity may account for a role of A-SMase in the pathogenesis of different diseases [40, 55–60]. We have now identified a novel mechanism of action of A-SMase in melanomas and found that the enzyme down-regulates the autophagic process *via* activation of the mTOR pathway. Sphingolipids play a role in autophagy: ceramide and sphingosine 1 phosphate are structural and functional components of autophagic flux [39, 40, 61–65] and sphingolipid micro-domain disturbances occur during defective autophagy [66].

The mTOR-related autophagy was shown to be a protective mechanism for melanoma cells [22]. Although autophagy has been reported to have opposite roles on anticancer treatments, either increasing or decreasing their efficacy depending on the cell type [18, 19, 23], a general agreement exists that its primary role is to enhance cancer cells’ resistance to chemotherapy treatment, and inhibition of autophagy may potentiate the re-sensitisation of therapeutic-resistant cancer cells to anticancer drugs [18, 20, 49, 67]. In this respect, in ongoing clinical trials, including those in melanoma, chloroquine is often used in combination with chemotherapeutic drugs, radiotherapy, some targeted therapies and immunotherapy [18, 21, 68, 69]. We demonstrate here that chloroquine treatment of melanoma-bearing mice enhances the response to cisplatin induced by low A-SMase while no effects were observed in tumours characterised by sustained A-SMase levels. These findings indicate that modulation of autophagy induced by A-SMase has a key role in melanoma chemo-resistance. In particular, the up-regulation of autophagic machine which occurs in melanoma cells expressing low A-SMase significantly decreases melanoma sensitivity to cisplatin, thus suggesting the ability of low A-SMase to promote autophagy, and hence tumour cell growth. Indeed, constitutive formation of autophagosomes was detected in invasive and metastatic melanoma cells [24] consistently with the fact that patients with lower number of autophagic vacuoles per melanoma cell are more responsive to chemotherapy and have better survival [70]. Interestingly, autophagy inhibition is required to restore cell death in melanoma cells resistant to immunotherapy [71]. Accordingly, it has been recently shown that the promotion of autophagy facilitates cisplatin resistance in melanoma cells [48]. Recently identified autophagy inhibitors appear to have the potential to sensitise melanomas to first-line therapies [72] and chemotherapy, including that with platinum derivatives [73].

Cisplatin represents one of the most successful drugs in chemotherapy leading to the discovery of several new generations of anticancer compounds which have become an invaluable tool in the oncologist's arsenal. Even though it is an “old” drug as the other chemotherapy agents, cisplatin continues to find uses in different types of cancers, especially as it is synergistic with other agents. In particular, in advanced melanoma traditional types of chemotherapy, including cisplatin-containing combination regimens, are still used although they do not longer represent the first-line therapy [4–8]. Cisplatin resistance of tumour cells has most often a multifactorial nature and involves different cellular mechanisms, as for instance the altered expression of proteins in signal transduction pathways that control apoptosis/autophagy [18, 74]. With respect to chemo-resistance to cisplatin in melanoma cells, the role of A-SMase in the modulation of apoptosis and autophagy (also induced by cisplatin) led us to hypothesise that low A-SMase, as occurs in more aggressive cells [38], result in a loss of chemotherapy response through both the inhibition of apoptosis and the activation of autophagy. A greater understanding of sphingolipid-mediated crosstalk between apoptosis and autophagy in melanomas may thus be critical for enhancing the chemotherapeutic efficacy of these agents [75]. In this respect, recent *in vitro* data emphasize the potential and advantages of manipulating sphingolipid system, apoptosis, and autophagy for glioma chemotherapy [76]. On the other hand it has been also reported that A-SMase overexpression in glioma cells does not enhance the anti-glioma activity of chemotherapy [77].

In conclusion, A-SMase is an attractive target in anti-tumour strategy for melanomas. Our data encourage pre-clinical testing on the up-regulation of A-SMase levels/activity as possible novel anti-neoplastic strategy to circumvent chemo-resistance, enhance the effects of chemotherapy and improve clinical outcomes.

## MATERIALS AND METHODS

### Animals

Experiments were performed on C57BL/6 female mice (Charles River Laboratories, Calco, Italy) at 6-8 weeks after birth (ca. 20 g body weight). Animals were kept in a regulated environment (23 ± 1°C, 50 ± 5% humidity) with a 12 h light/dark cycle (lights on at 08.00 a.m.) and fed *ad libitum*. All studies were conducted in accordance with the Italian law on animal care N° 116/1992 and the European Communities Council Directive EEC/609/86. The experimental protocols were also approved by the Ethics Committee of the University of Milano. All efforts were made to reduce both animal suffering and the number of animals used.

### Cell cultures

In agreement with published protocols [37, 38], murine melanoma B16 cells and human melanoma cells Det-mel and MSR3 were cultured in Iscove's supplemented with 10% heat-inactivated foetal bovine serum (FBS), glutamine (200 mM), penicillin/streptavidin (100 U/ml), 1% Hepes 1M pH 7.4 and grown at 37°C in a humidified atmosphere containing 5% CO_2_. The low expressing A-SMase B16-W6_pSIL10 and the high expressing A-SMase B16-F1_B1A clone were generated as previously described [37, 38]. Previous results indicated that the transfection of B16-W6 cells with the plasmid harbouring a shRNA containing a scrambled sequence (B16-W6_scr, used as control for B16-W6_pSIL10) or B16-F1 cells with the empty vector pEF1/Myc (used as control for B16-F1_B1A) did not modify unspecifically the cell content/activity of A-SMase, the cell phenotype and growing properties [37, 38].

### Animal handling and allograft tumour model

On day 0, mice were injected sub-cutaneously with the tumourigenic dose of 2.5 × 10^4^ B16-W6_scr, B16-W6_pSIL10, or B16-F1_B1A cells, in the lower-right flank [35, 37, 38]. Before the injection, melanoma cells were controlled for their levels of expression of A-SMase by western blotting. When the syngeneic tumour implantation was established (usually 10 days after tumour cells inoculation) and the tumour was palpable, transplanted mice received 100 μl intraperitoneal injections of cisplatin (4 mg/kg) [35] or vehicle (phosphate buffer saline - PBS) three times every other day. When indicated, B16-W6_pSIL10 and B16-F1_B1A transplanted mice also received 15 μl injection in the peritumoural area of chloroquine diphosphate (30 mM) [49, 51] or vehicle (PBS). Tumour growth was monitored every 2-3 days by means of external calliper measurements and volume calculation (length x width^2^/2), until mice reached IACUC euthanasia criteria, as for instance clinical signs of tumour or when tumour size exceeded 10% of body weight (ca. 1500 mm^3^ tumour volume) [35, 37, 38]. The mice were also observed to determine the duration of survival for each group using the Kaplan-Meier estimator (median survival). When indicated, mice were sacrificed when tumour size reached ca. 500 mm^3^ volume, and tumour collected for further analysis (*i.e.* TUNEL assay, Transmission electron microscopy analysis, and fluorescence microscopy).

### TUNEL assay

The collected melanoma transplants were fixed in ice cold 4% paraformaldehyde before being rinsed in PBS and cryo-protected overnight in 30% sucrose. Tissues were then embedded in O.C.T. Compound (Sakura, AJAlphen aan den Rijn, The Netherlands) and cut in a CM1850 UV cryostat (Leica Biosystems, Wetzlar, Germany). At least 5 cryosections (6 μm) were obtained from each tumour and assayed for apoptosis by the TUNEL method (DeadEnd Fluorometric TUNEL System; Promega, Milano, Italy), according to the manufacturer's protocol. After DAPI counterstaining, samples were mounted with Vectashield (Vector Laboratories, Burlingame, CA, USA) and examined using a DMI4000 B automated inverted microscope equipped with a DCF310 digital camera (Leica Microsystems, Wetzlar, Germany). Image acquisition was controlled by the Leica LAS AF software.

### Flow cytometry

Apoptotic mouse and human melanoma cells were evaluated by flow cytometry as described previously [38, 78–81]. Cells were incubated with 1 μg/ml Annexin V-FITC (to assess the phosphatidylserine exposure on the outer leaflet of the plasma membrane) and 2 μg/ml PI (DNA-binding probe) for 15 min at 4°C before being analysed by flow cytometry using Gallios Flow Cytometer (Beckman-Coulter, Brea, CA, USA) and the software FCS Express 4 (De Novo System, Portland, OR, USA).

### MTT assay

Using published protocols [38, 80, 82–84], cell viability of mouse and human melanoma cells was evaluated by MTT analysis. Each experimental condition was replicated in 8 wells. Cells were then washed and fresh medium containing MTT (0.5 mg/ml) was added in each well. After 4 h incubation at 37°C, the supernatant was gently removed and formazan crystals were dissolved in DMSO. Absorbance was recorded at 570 nm with correction at 690 nm using a Glomax Multi Detection System microplate reader (Promega, Milano, Italy).

### A-SMase activity assay

As previously published [37], cells were homogenised in the acid lysis buffer (50 mM sodium acetate, 1% Triton X-100, 1 mM EDTA, pH 5) with freshly added protease inhibitor cocktail (cOmplete; Roche Diagnostics, Milano, Italy). Sphingomyelinase activity was measured using the Amplex Red Sphingomyelinase Assay Kit (Life Technologies, Monza, Italy), as described in the manufacturer's protocol.

### Protein isolation and western blotting

Mouse melanoma cells were homogenised for 10 min at 4°C in RIPA lysis buffer, containing 50 mM Tris-HCl (pH 7.4), 150 mM NaCl, 1% NP-40, 1% sodium deoxycholate, 1 mM EDTA and 0.1% sodium dodecyl sulphate (SDS). Buffers were supplemented with a cocktail of protease and phosphatase inhibitors (cOmplete and PhosSTOP; Roche Diagnostics, Milano, Italy). Protein concentration was determined using the Bicinchoninic acid assay (ThermoFisher Scientific, Waltham, MA, USA). SDS and β-mercaptoethanol were added to samples before boiling, and equal amounts of proteins (40 μg/lane) were separated by 4-20% SDS-polyacrylamide gel electrophoresis (Criterion TGX Stain-free precast gels and Criterion Cell system; Bio-Rad, Hercules, CA, USA). Proteins were then transferred onto nitrocellulose membrane using a Bio-Rad Trans-Blot Turbo System. The membranes were probed using the following primary antibodies: rabbit polyclonal anti-LC3B, anti-p62/SQSTM1 (Sigma-Aldrich, Saint Louis, MO, USA), anti-phospho-Akt (Ser473), anti-phospho-S6 ribosomal protein (Ser240/244), anti-phospho-Ulk1 (Ser757), and rabbit monoclonal anti-cleaved caspase 3 (Cell Signaling Technology, Danvers, MA, USA). After the incubation with the appropriate horseradish-peroxidase (HRP)-conjugated secondary antibody (Cell Signaling Technology, Danvers, MA, USA), bands were visualised using the Clarity Western ECL substrate with a ChemiDoc MP imaging system (Bio-Rad, Hercules, CA, USA). To monitor for potential artefacts in loading and transfer among samples in different lanes, the blots were routinely treated with the Restore Western Blot Stripping Buffer (ThermoFisher Scientific, Waltham, MA, USA) and re-probed with the rabbit polyclonal anti-GAPDH (FL-335) primary antibody (Santa Cruz Biotechnology, Dallas, TX, USA). When appropriate, primary rabbit polyclonal Akt, monoclonal S6 ribosomal protein (54D2), and monoclonal Ulk1 (D8H5) antibodies (Cell Signaling Technology, Danvers, MA, USA) that recognise the protein independently of its phosphorylation state were also used in reprobing experiments. Bands were quantified for densitometry [52] using the Image Lab software (Bio-Rad, Hercules, CA, USA).

### Transmission electron microscopy

The collected melanoma transplants were reduced into smaller blocks and stored overnight at 4°C in a fixative solution containing 2% formaldehyde and 2% glutaraldehyde in 0.1 M sodium cacodylate buffer, pH 7.3. Fixed specimens were washed in cacodylate buffer and postfixed at 0°C for 1.5 h in 2% osmium tetroxide. The samples were washed in distilled water, stained in block in 2% aqueous uranyl acetate, dehydrated through an ascending series of ethanol and embedded in Araldite resin. For ultrastructural observations at least 5 ultra-thin sections (60-90 nm) were obtained from each tumour. Sections were collected on 100-mesh grids, counterstained with lead citrate and photographed (magnification 2500 x) with a EM 10 electron microscope (Carl Zeiss, Oberkochen, Germany). Micrographs were scanned in a flat-bed scanner and images were merged.

### Fluorescence microscopy

Using published protocols for immunofluorescence of melanoma transplants [38], 6 μm cryosections were treated with citrate buffer for antigen retrieval. Tissue auto-fluorescence was quenched with 0.1 M glycine-PBS for 10 min. Sections were then incubated with blocking solution containing 10% goat serum and 1% BSA (30 min) before 1 h staining with the rabbit polyclonal anti-p62/SQSTM1 primary antibody (Sigma-Aldrich, Saint Louis, MO, USA) in blocking solution supplemented with 0.1% saponin. For fluorescent detection Alexa Fluor 546 dye-conjugated anti-rabbit IgG (Life Technologies, Monza, Italy) was used. Tissue sections were rinsed and coverslips added in the mounting medium containing DAPI. At least 5 tissue sections were obtained from each tumour. Images were acquired with a DMI4000 B automated inverted microscope as specified above. Immuno-reactivity was quantified with ImageJ software (http://rsbweb.nih.gov/ij/). In particular, the extent of staining of each section was calculated as integrated optical density, which is equal to the area × average density of image occupied by immune-reactivity and represented in graph.

### Statistics

Tumour growth was analysed using two-way ANOVA, followed by the Bonferroni post-test. pEC_50_ was determined by non-linear regression curve analysis of the concentration-effect responses. Differences in pEC_50_ values among concentration-response curves were calculated with the F test. For the other experiments, statistical significance of data between the groups was evaluated using unpaired Student's *t*-test (single comparisons) or one-way ANOVA followed by the Newman-Keuls post-test (multiple comparisons). When indicated, data belonging from different experiments were represented and averaged in the same graph. The GraphPad Prism software package (Graph Software, San Diego, CA, USA) was used. The results were expressed as means ± SEM of the indicated n values.

### Chemicals

Iscove's modified Dulbecco's medium, Hepes, FBS, PBS, glutamine, penicillin/streptavidin, were purchased from Euroclone (Pero, Italy). Cisplatin (Cisplatino Teva) was from Teva Pharma Italia (Milano, Italy). Human A-SMase (Recombinant Human SMPD1, His-tagged) was purchased from Creative BioMart (Shirley, NY, USA). Annexin V-Fluorescein Isothiocianate (FITC) and PI were obtained from Life Technologies (Monza, Italy) and eBioscience (San Diego, CA, USA), respectively. All other reagents were purchased from Sigma-Aldrich (Saint Louis, MO, USA).

## SUPPLEMENTARY MATERIAL FIGURES


