# Supplementary material for: Essential role for acid sphingomyelinase-inhibited autophagy in melanoma response to cisplatin
Source: Oncotarget. 2016 Apr 14;7(18):24995–5009. doi: 10.18632/oncotarget.8735 (PMC5041885; doi:10.18632/oncotarget.8735)
Supplement: Supplementary file 1 [file oncotarget-07-24995-s001.pdf]

Essential role for acid sphingomyelinase-inhibited autophagy in melanoma response to cisplatin

Supplementary Material

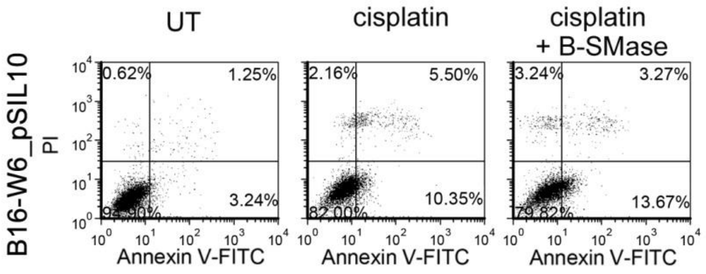

Supplementary Figure 1: B1-W6_pSIL10 were cultured for 16 h in the absence (UT: untreated) or in the presence of cisplatin (10 µg/ml) and cisplatin + bacterial A-SMase (B-SMase; 0.3 units/ml). Representative dot plots of Annexin V-FITC/PI staining were shown**.** The images are representative of results obtained from three experiments.

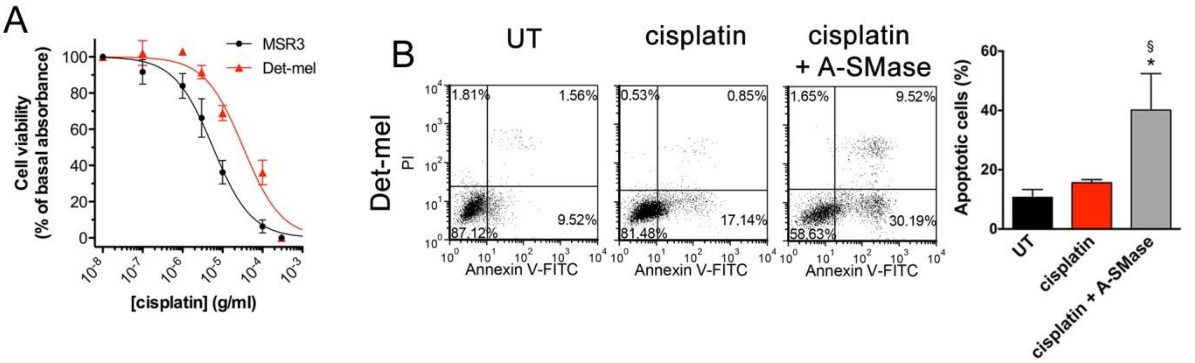

Supplementary Figure 2: A. Dose-response curves of the effects of cisplatin on the viability of MSR3 and Det-mel cells, as measured by the MTT assay (n = 3). **B.** Det-mel cells were cultured for 16 h in the absence (UT: untreated) or in the presence of cisplatin (10 μg/ml), both alone or in combination with human A-SMase (2.0 U/ml) (n = 4). Left panels: representative dot plots of Annexin V-FITC/PI staining; Right panel: apoptosis quantification expressed as percentage of apoptotic cells (Annexin V^+/PI^- and Annexin V^+/PI^+ cells). Statistical significance * and § $p < 0.05$ *vs* UT and cisplatin-treated cells, respectively.
